# Effects of Composition and Polymerization Conditions on the Electro-Optic Performance of Liquid Crystal–Polymer Composites Doped with Ferroelectric Nanoparticles

**DOI:** 10.3390/nano14110961

**Published:** 2024-05-31

**Authors:** Gaby Nordendorf, Gisela Jünnemann-Held, Alexander Lorenz, Heinz-Siegfried Kitzerow

**Affiliations:** 1 Department of Chemistry, Faculty of Science, Paderborn University, Warburger Str. 100, 33098 Paderborn, Germany; gaby.nordendorf@uni-paderborn.de (G.N.); gisela.juennemann@uni-paderborn.de (G.J.-H.); alexander.lorenz@online.ms (A.L.); 2 Center for Optoelectronics and Photonics Paderborn (CeOPP), Paderborn University, Warburger Str. 100, 33098 Paderborn, Germany; 3 COVDEAG-CCO-SF-GV-AD, Covestro Deutschland AG, 51365 Leverkusen, Germany

**Keywords:** polymer network, liquid crystal, ferroelectric nanoparticles, phase transition temperature, electro-optic switching

## Abstract

The presence of a polymer network and/or the addition of ferroelectric nanoparticles to a nematic liquid crystal are found to lower transition temperatures and birefringence, which indicates reduced orientational order. In addition, the electro-optic switching voltage is considerably increased when a polymer network is formed by in situ polymerization in the nematic state. However, the resulting polymer network liquid crystal switches at similar voltages as the neat liquid crystal when polymerization is performed at an elevated temperature in the isotropic state. When nanoparticle dispersions are polymerized at an applied DC voltage, the transition temperatures and switching voltages are reduced, yet they are larger than those observed for polymer network liquid crystals without nanoparticles polymerized in the isotropic phase.

## 1. Introduction

In a liquid crystal (LC) [1], the dispersion of nanoparticles (NPs) can change the optical and electro-optic behavior considerably [2]. In principle, the presence of NPs can shift phase transition temperatures or change the LC order parameter at a given temperature [3]. In addition, there are specific influences by special kinds of NPs. For example, porous NPs made of a silica aerogel may provide an optically addressable storage effect thereby facilitating the writing and selective erasing of optical information by a laser beam [4]; gold NPs can add optical effects that are based on plasmonic resonances [5], changes in LC surface anchoring, or enhanced conductivity [6]; semiconductor nanoparticles can equip the composite with luminescence [7]; and magnetic NPs [8] and ferroelectric NPs [3,9] can enhance sensitivity to external magnetic and electric fields, respectively. Also, composites consisting of a low molar mass LC and a polymer can show properties that differ dramatically from the properties of pure LC. Composites with a large polymer content consist of isolated LC droplets embedded in a polymer film. These systems are known as nematic curvilinear aligned phases (NCAPs) [10] or polymer-dispersed liquid crystals (PDLCs) [11,12,13], depending on their method of preparation. They may be used to fabricate electrically addressable flexible films, which can be switched, for example, between a transparent and a translucent state [10,11,12,13]. In contrast, LC/polymer composites with interconnected LC regions or liquid crystal films pervaded by a fine network of interconnected fibers [14,15] are referred to as anisotropic gels [15], polymer-stabilized liquid crystals (PSLCs) [16,17,18,19], polymer network liquid crystals (PNLCs) [20,21], or polymer network-stabilized liquid crystals [22]. Applications of such composites with a low polymer fraction utilize the stabilizing effect of an anisotropic polymer network (typically formed by the in situ photopolymerization of reactive mesogens) on the LC orientation [14,15], on one of several different switching states (for example, polymer-stabilized cholesteric texture, PSCT [16]), or on the temperature range of the thermal stability of a particular mesophase (for example, the polymer-stabilized blue phase, PSBP [17]). Copolymer network LCs (which are formed when the LC is doped with a mesogenic and non-mesogenic photo-reactive monomer) may be beneficial to yield unusually small switching times (*τ_on_* + *τ_off_*) < 3 ms [23]. In general, LC/polymer composites can be used for large-area smart windows, flexible displays, large-area bistable displays, reflective displays, displays without any alignment layer, polarizer-free displays, and other purposes. Many applications are feasible, owing to the huge variety of LC mesophases and the versatility of network morphologies [24]. In addition to different electro-optic properties of the LC, varying properties of the composites depending on the ratio of LC and polymer contents, varying orientational orders of the polymer, and different crosslinking densities, the conditions during polymer formation can influence the network morphology and the resulting behavior of the composites dramatically [25,26,27,28,29,30,31,32]. The stabilization of an LC by a polymer network formed in the field-off state is well-known to result in higher driving voltages. Yet, a decrease in the driving voltage because of enhanced curing temperature was found by other researchers [25,26,27,28,29,30] and attributed to either larger pore sizes [25] or a lower fractal dimension of the network formed at elevated temperatures [29,30]. A voltage applied during the curing process was found in previous works to promote fast switching [31] or to imprint a bias effect on the switching behavior [32]. Combining both nanoparticle doping and polymer networks in LCs is currently a topical field of research [33,34,35,36].

The present work is motivated by previous studies of the versatile interactions of liquid crystals with iron-doped lithium niobate (LiNbO_3_:Fe) crystals [37,38]. The latter material does not only exhibit ferroelectric properties but also an anomalous photovoltaic effect. Local illumination of a LiNbO_3_:Fe substrate can induce very high electric field strengths (rather than electric current), which in turn may reorient a neighboring liquid crystal, thereby facilitating optical writing, the selective erasing of information, or optical vortex generation owing to defect appearance [37,38]. Surface-grafted photo-initiators based on a silane moiety have been synthesized in order to enable the in situ formation of a polymer network that is covalently bound to a LiNbO_3_:Fe surface, aiming to combine the opportunities of the anomalous photovoltaic effect with the enhanced stability or modified electro-optic response of an LC/polymer network composite [39]. More recently, this work was extended to LC dispersions, where the LC contains LiNbO_3_:Fe nanoparticles rather than being confined by a monocrystalline LiNbO_3_:Fe substrate [40]. This preceding study revealed that the surface effect of the silane compounds is also beneficial to the milling process, in which LiNbO_3_:Fe nanoparticles are fabricated from larger LiNbO_3_:Fe crystals. Adding these silanes to the respective grinding mixture yields sufficiently small particles with a limited size distribution [40]. Here, the effects of either dispersing ferroelectric LiNbO_3_:Fe nanoparticles in a liquid crystal matrix or adding a stabilizing cross-linked polymer to the same liquid crystal matrix are investigated in greater detail. The resulting changes in the transition temperatures and the electro-optic performance of the different samples are studied. The PSLC composites used in this study are formed by in situ photopolymerization under different conditions, i.e., either in the nematic mesophase at room temperature or in the isotropic phase at a temperature above the clearing point, and are photo-cured either without an applied voltage or with an applied dc voltage. In addition to confirming the influence of these different polymerization conditions on the properties of the resulting PNLCs, we extend hitherto knowledge to the thermal and electro-optic behavior of PNLC/ferroelectric NP nanocomposites. For this purpose, we modified a recently developed protocol for fabricating monodisperse, LC-dispersible ferroelectric NPs [40]. Because of a higher rotational speed during the grinding process and filtration, stable dispersions of particles smaller than 200 nm could be studied.

## 2. Materials and Methods

The well-known nematic LC mixture E7 (Merck, Darmstadt) was used as a matrix, which consists of para-substituted cyano-alkyl-biphenyls and terphenyls [41] and exhibits a nematic phase at room temperature. The mixture E7 is commercially available and has been widely studied, so its material properties can be found in the literature. In addition to the pure LC, dispersions of LiNbO_3_:Fe nanoparticles (NPs) in the same liquid crystal [in short: LC/NP dispersions, Table 1], polymer network liquid crystals (PNLCs, Table 1), and LC composites containing both 1% (by weight) NPs and a polymer network (PNLC/NP dispersions, Table 1) were investigated. The NPs and the composites were prepared as follows.

### 2.1. Preparation of Nanoparticles and Dispersions of the Particles in the Liquid Crystal

**Figure 1:**
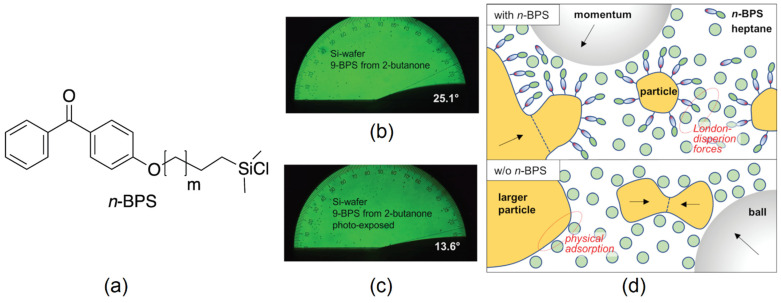
Effects of surface-grafted photo-reactive surfactants *n*-BPS on the grinding process yielding LiNbO_3_:Fe nanoparticles and on the surface tension of a solid substrate [39]. (**a**) Molecular structure of *n*-BPS, with *n* = *m* + 2 indicating the length of the alky chain that separates the surface-active silane group from the photo-initiating benzophenone moiety. (**b**,**c**) Contact angle of the LC E7 on Si wafer surfaces coated with *9*-BPS (**b**) before and (**c**) after UV exposure. (**d**) Presumable interactions of colloidal particles during the ball milling process with *n*-BPS (**above**) and without *n*-BPS (**below**). Parts of this figure were published earlier in Ref. [40] (© Royal Society of Chemistry).

Nanoparticles made of iron-doped lithium niobate (LiNbO_3_:Fe) were fabricated by grinding from LiNbO_3_:Fe single crystal substrates (0.05 mole-% Fe, purchased from Deltronic Crystal Industries Inc.) using a planetary ball mill, type PM 200 from Retsch GmbH, Haan, Germany. According to the general instructions for colloidal grinding provided by Retsch, a dispersion of the solid in 5 mL ethanol was ground for three hours at 500 rpm. Based on experiences with the preparation of ferroelectric NPs made of BaTiO_3_ [9], a surfactant was added during the milling process to achieve particles that can be homogeneously dispersed in an LC. Here, surface-grafting benzophenone silane derivatives (as described in Ref. [40]) were used for this purpose. The molecules of this novel type of surfactant (*n*-BPS) [inset of Figure 1a] contain two functional groups, a chlorosilane anchor group and a photo-chemically active benzophenone group, which are separated by an alkyl chain with *n* = *m* + 2 carbon atoms. In earlier studies, they were confirmed to bind covalently to solid substrates if a catalytic amount of triethylamine is added and to change the contact angle of wetting fluids when the surface is exposed to UV radiation (Figure 1b,c). Comparing Figure 1b and Figure 1c, a decrease in the contact angle is observed for the UV-exposed samples. This result reveals that LC molecules were successfully attached to the surface in the polymerization process, which is expected to reduce surface tension [40]. In addition, the surface-grafting agents were found to yield smaller particles in the ball milling process than oleic acid at reasonable milling times. The latter benefit can be attributed to their covalent bonding to NP surfaces (Figure 1d), which prevents NPs from reconglomerating during the milling process [40]. The particle size distribution was obtained from light scattering experiments on particles dispersed in ethanol. The results indicate particle sizes of only a few hundred nanometers. To ensure that no particles > 200 nm were added to the composite, the dispersion was passed through a PTFE filter, and the NPs were dried before adding them to the composite mixture. More details are given in Ref. [40]. After measurements of the size distribution, the particles were dispersed in the LC mixture E7, yielding an LC/NP dispersion containing 1% by weight of the nanoparticles (Table 1).

### 2.2. Preparation of Polymer-Stabilized Liquid Crystals

Polymer-stabilized liquid crystals were obtained by mixing the nematic liquid crystal mixture E7 with the non-mesogenic photo-reactive monomer EHA, the mesogenic photo-reactive monomer RM257, and the photo-initiator Irgacure 819 (IRG 819 from Ciba Specialty Chemicals Inc, Basle, Switzerland). The concentrations of the components are given in Table 1. Electro-optic test cells from E. H. C. Inc. (Tokyo, Japan) were filled with this photosensitive mixture to obtain a uniformly aligned nematic film. The test cells consist of two glass substrates equipped with transparent electrode areas (4 mm × 4 mm) made of indium tin oxide (ITO) and uniformly rubbed alignment layers made of polyimide (PI). The spacing between the two substrates of the cells is *d* = 2 µm. The cell thickness was chosen to be sufficiently small to avoid optical retardation between the ordinary and the extraordinary light beam by more than one wavelength, which would cause multiple intensity maxima and minima on increasing voltage (cf. Equation (1) in Section 3.2). After filling the cell through capillary forces, the samples were allowed to rest until a uniformly aligned nematic film was observed. Subsequently, the samples were cured by irradiating with near-ultraviolet (UV-A) radiation at a power density of 3 mW/cm^2^ for three minutes. Consequently, a uniformly aligned polymer-stabilized nematic film was obtained. Some of the samples were heated to 65 °C (i.e., to a temperature above the clearing point of E7) and photopolymerized in the isotropic phase of E7 at this elevated temperature in order to investigate the influence of the curing temperature on the phase transition temperatures and the electro-optic performance of the resulting PSLC composite.

### 2.3. Preparation of Polymer-Stabilized Liquid Crystal/Nanoparticle Dispersions

In addition to the pure liquid crystal E7, LC/NP dispersions, and PSLCs based on E7, E7-based PSLC/NP composites were prepared that contain both a polymer network and NPs (Table 1). In the latter case, NPs were fabricated by planetary ball milling, as described in Section 2.1, and thoroughly dispersed in a photo-sensitive precursor mixture (as described in Section 2.2). Subsequently, an electro-optic test cell from E. H. C. was filled with this photo-reactive NP dispersion, the nematic dispersion was allowed to align uniformly, and subsequently, the sample was cured by UV radiation (UV-A, 3 mW/cm^2^, 3 min). Some samples were cured at an elevated temperature (65 °C) at which the composite exhibits an isotropic liquid phase. In addition, a sample was cured while a DC voltage above the threshold voltage of the Fréedericksz effect was applied during the photopolymerization process.

### 2.4. Thermal and Electro-Optic Characterization of the Samples

**Figure 2:**
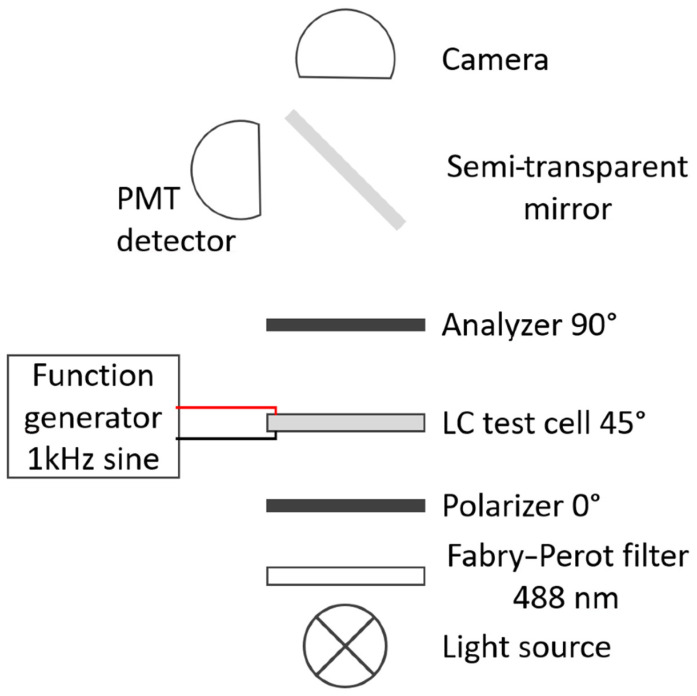
Experimental setup that was used to observe the mesophases appearing in different temperature ranges and to measure the transition temperatures. The specimen was placed between crossed polarizers in a polarizing microscope and observed in transmission. For thermal characterization, the sample was illuminated with white light (i.e., without a Fabry–Perot filter), and the textures were recorded by a CCD camera while the temperature was controlled using a commercial hot stage. The same setup was used to study the electro-optic response at room temperature. In the latter case, however, the sample was illuminated with monochromatic light (at the wavelength 488 nm, using a color filter), and the transmitted light intensity was measured using a photomultiplier tube (PMT).

The transition temperatures of the samples were measured by observing the appearance of the samples among crossed polarizers using polarized optical microscopy (POM, Figure 2). The temperature of the samples was varied and controlled using a commercially available microscope hot stage (Linkam). The isotropic phase observed in transmission using white light appears dark when the sample is placed between crossed polarizers. In contrast, the nematic phase changes the state of polarization of the transmitted light, owing to its birefringence, and appears bright under these conditions.

For electro-optic characterization, commercially available test cells from E. H. C. (as described in Section 2.2) were filled with E7 or the respective composite. The PI alignment layers, rubbed at an azimuthal angle of *φ*_0_ = 0° provided a uniform alignment of the LC director (i.e., the optical axis of the uniaxial LC) along the rubbing direction. The specimens were observed via POM in transmission mode using monochromatic light (488 nm) and crossed polarizers (Figure 2). The respective sample was rotated on a rotating stage to find the dark state, which appears when the director is aligned parallel or perpendicular to the plane of polarization of the incident light. Subsequently, the sample was rotated around the surface normal so that the angle between the LC director and the electric field of the incident light was adjusted to *φ* = 45°. This azimuthal angle corresponds to the maximum transmitted intensity that is possible for the given optical retardation of the sample. The amplitude of an applied AC voltage was linearly varied, and the intensity of the transmitted light was measured and recorded as a function of the root mean square (rms) value of the applied voltage. Since the liquid crystal E7 exhibits positive dielectric anisotropy, the LC director aligns gradually along the quasi-static electric direction (perpendicular to the substrates) so that the effective birefringence decreases with increasing amplitude of the voltage (Fréedericksz transition) [1,42]. This effect may lead to nonlinear changes in the transmitted light intensity until the sample finally becomes dark in the limit of very high field strength.

## 3. Results

### 3.1. Phase Transition Temperatures of the Composites

**Figure 3:**
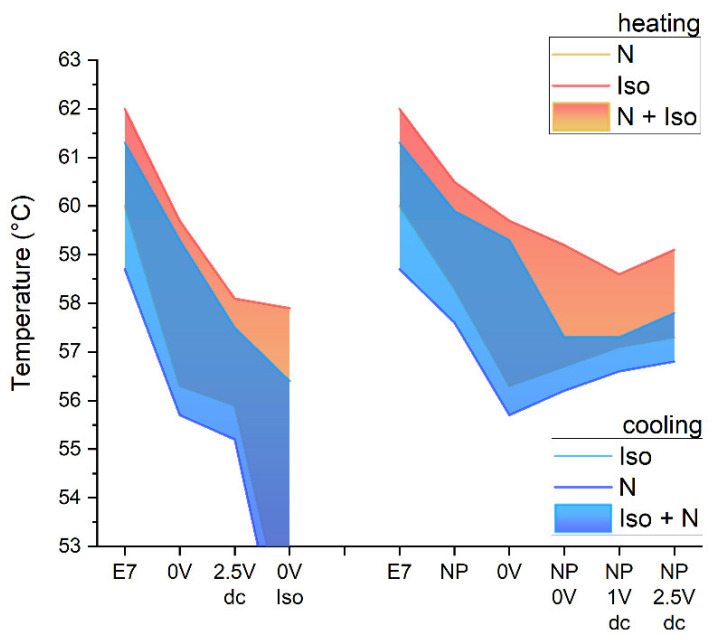
Phase transition temperatures for E7, PNLC samples (to the (**left**)), and PNLC samples containing nanoparticles (to the (**right**)) polymerized under various conditions. Areas extending to temperatures below 53 °C indicate that the lower limit of the nematic–isotropic coexistence could not be determined by POM textures for the sample that was polymerized at 0 V in the isotropic state.

An overview of the effects of a polymer network and the addition of nanoparticles on the mesomorphic behavior of E7 is given in Figure 3. All composites investigated in this study show a nematic phase yet reduced transition temperatures with respect to the pure liquid crystal mixture (“E7” in Figure 3). The clearing temperatures on cooling (blue) are typically 0.5–1 °C smaller than the clearing temperatures on heating. Close to the clearing temperature, the nematic phase and the isotropic phase coexist, typically within a range of a few centigrade. In the samples without nanoparticles (displayed to the left in Figure 3), the sample containing a polymer network cured in the planar-aligned state at room temperature without an applied field (“0 V” in Figure 3) shows transition temperatures reduced by only 2 °C with respect to pure E7. In contrast, the sample cured at an applied voltage of 2.5 V (labeled “2.5 V dc” in Figure 3) shows much larger reductions in the transition temperatures (by about 4 °C). Presumably, this effect can be attributed to the aligning effect of the applied voltage. The quasi-static electric field tends to align the director perpendicular to the substrate. Consequently, the orientation of the long axis of the mesogenic crosslinking moieties (formed from RM257 molecules) may no longer match the uniform planar orientation of the E7 molecules. Accordingly, the orientational order of the mesophase is destabilized rather than being stabilized by the polymer network, which results in reduced thermal stability of the nematic phase. A similar effect is observed when the reactive precursor mixture is heated above the clearing temperature and cured in the isotropic phase (“0 V Iso” in Figure 3). Again, the clearing temperature is reduced by about 4 °C. Additionally, the stabilization of the isotropic phase by the polymer network in this latter case increases the temperature range of isotropic/nematic coexistence to the extent that small isotropic islands remain in the nematic phase even at room temperature. Adding 1% NPs to E7 (“NP” in Figure 3) or to the PSLC (“NP 0 V” in Figure 3) reduces the clearing temperature in comparison with the respective sample without nanoparticles only slightly (by about 1 °C), which indicates a small perturbation of the orientational order by the NPs. A similar effect is observed when NP/PSLC dispersions are cured at 1 V or 2.5 V (“NP 1 V dc” and “NP 2.5 V dc” in Figure 3, respectively). The sample “0 V Iso” (without NPs cured in the isotropic phase) and the samples containing both NPs and a polymer network differ from the other samples by a larger hysteresis of the clearing temperatures. Obviously, isotropic inclusions in the polymer network (either by isotropic islands or by NPs) promote super-cooling of the isotropic phase at the isotropic–nematic phase transition.

### 3.2. Electro-Optic Properties

**Figure 4:**
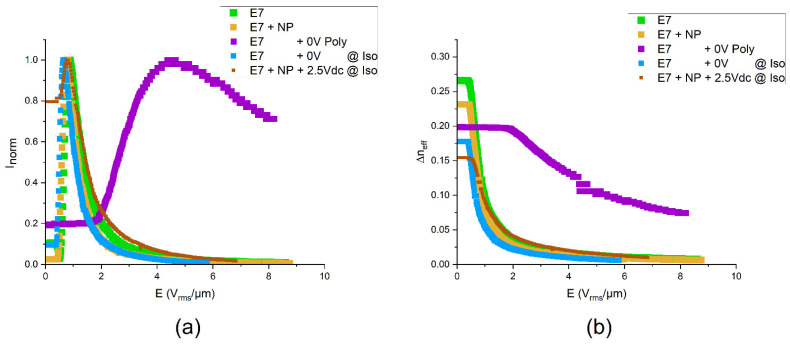
Electro-optic properties. (**a**) Intensity versus voltage and (**b**) effective birefringence versus voltage for Fréedericksz cells containing the liquid crystal mixture E7, different fractions of ferroelectric LiNbO_3_:Fe nanoparticles, and different contents of a polymer network that was formed at various conditions.

The electro-optic responses of E7 and its composites are represented in Figure 4a, where the normalized transmitted light intensity *I_norm_* is plotted against the root mean square values of the applied electric field *E*. As expected, the intensity versus voltage shows minima and maxima as soon as the voltage exceeds a threshold value. In the limit of very large voltages, the intensity vanishes. This behavior agrees with the well-known Fréedericksz transition, i.e., a field-induced reorientation of the LC director from an initial alignment parallel to the substrate to an alignment parallel to the electric field (i.e., perpendicular to the substrate). In comparison with the pure liquid crystal (“E7” in Figure 4), a nanoparticle dispersion without polymer (1 wt.% NPs, “E7 + NP” in Figure 4) shows a slightly reduced threshold voltage and slightly reduced operation voltages. In contrast, the threshold field strength and the field strength of the intensity maximum are strongly increased (roughly by a factor of 4) when the LC is stabilized by a polymer network formed in situ at room temperature without applied voltage (“E7 + 0 V Poly” in Figure 4). This striking effect demonstrates the strong effect of the polymer network, which—in this case—stabilizes the field-off state of the director field, thereby impeding the field-induced orientation. However, when the polymer network is formed at elevated temperature in the isotropic liquid phase (“E7 + 0 V @ Iso” in Figure 4), the threshold voltage and the voltages yielding equivalent intensities are even reduced in comparison with pure E7. Finally, a sample containing both NPs and a polymer network prepared in the isotropic state at an applied voltage responds to the external field at voltages similar to those of the pure liquid crystal.

In more detail, the electro-optic characteristics can be analyzed as follows. A uniformly aligned planar Fréedericksz cell behaves like an optical retarder. The transmitted intensity of polarized light can be calculated by the Jones formalism or the Müller–Stokes formalism [43]. For a cell placed between crossed polarizers, the transmitted intensity behind the analyzer (i.e., second polarizer) is given by

*I*(*V*) = ½ *I*_0_ sin^2^(2*φ*) sin^2^{π Δ*n_e,eff_*(*V*) *d*/λ}, (1)

where *I*_0_ is the initial before passing the first polarizer, φ is the azimuthal angle between the optical axis of the retarder and the plane of polarization of the incident light, *Δn_eff_* = *n_e,eff_*(*V*) − *n_o_* is the effective birefringence, *d* is the thickness of the birefringent layer, and *λ* is the wavelength of the monochromatic light. In the field-off state of the Fréedericksz cell, the nematic director is uniformly aligned parallel to the substrate, i.e., the polar angle *ϑ* between the optical axis and the direction of light propagation is *ϑ* = π/2. In this case, the effective birefringence Δ*n_eff_* exhibits its maximum value Δ*n* = *n_e_* − *n_o_*, where *n_e_* and *n_o_* are the extraordinary refractive index and the ordinary refractive index of the LC, respectively. When a voltage *V* is applied, the local director tends to be aligned along the field direction. The polar angle *ϑ* can be replaced by an effective value *ϑ_eff_*, which gradually decreases with increasing voltage. Consequently, the effective value of the extraordinary refractive index is

*n_e,eff_*(*V*) = {n_e_^−2^ sin^2^[*ϑ*_eff_(V)] + n_o_^−2^ cos^2^[*ϑ*_eff_(V)]}^−1/2^, (2)

and thus, the effective birefringence Δ*n_eff_* in Equation (1) decreases. For very high field strength, the effective extraordinary refractive index Δ*n_eff_* approaches the ordinary refractive index and the effective birefringence vanishes.

The dependences of the effective birefringence on the applied electric field strength calculated from the data shown in Figure 4a using Equation (1) are given in Figure 4b. This diagram shows the monotonous decrease in Δ*n_eff_* with increasing field strength. In addition to the voltages that are necessary to achieve a certain optical state for the different composites, Figure 4a also indicates the absolute values of the effective birefringence in the field-off state that indicate the relation of the orientational order parameters. It can be seen that pure E7 exhibits the highest birefringence in the field-off state, i.e., the highest order parameter. This value decreases when NPs are added, indicating that the latter disturb the orientational order slightly. Also, the PSLC formed at room temperature without an applied field shows a reduced birefringence. This observation may be attributed to using not only a reactive mesogen but a mixture containing also a non-mesogenic reactive compound (EHA). Not surprisingly, the formation of the polymer network in the isotropic phase reduces the orientational order further. If both NPs and a polymer network formed in the isotropic phase are acting against a uniform alignment of the LC molecules, the lowest effective birefringence is observed in the field-off state. Apart from these academic insights, it should be emphasized that a reduced birefringence is not necessarily disadvantageous. The sample thickness of 2 µm is close to the lower limit of cell gaps that can be manufactured reliably over large areas. Thus, for a Fréedericksz cell showing a monotonous decrease in the transmitted intensity versus voltage (without intermediate intensity minima and maxima), an even lower birefringence in the field-off state than the lowest value observed here would be beneficial. Inspection of the cells after one or two weeks revealed stability of the cells, i.e., no changes in the performance that would indicate ripening or degradation were found. The aging behavior of the materials over long periods or when exposed to different environmental conditions cannot be excluded, though.

## 4. Discussion and Conclusions

In summary, the results of this work pave the way towards complex composites that contain both ferroelectric nanoparticles and a polymer network. Reduced clearing temperatures and reduced birefringence of the composites in the field-off state indicate that both adding nanoparticles (NPs) to the LC mixture E7 and the in situ formation of a polymer network in E7 may reduce the orientational order in comparison with the pure LC. In the case of the polymer network, this effect may be attributed to the fact that the reactive precursor mixture contained a non-mesogenic compound. However, copolymerization of mesogenic and non-mesogenic reactive compounds was found to promote fast switching times in PSLC systems [29,30]. More importantly, the conditions of the in situ formation of the polymer network were found to affect electro-optic performance dramatically. While polymerization at room temperature in the field-off state requires very high driving voltages, PSLCs polymerized in the isotropic state show switching at similar voltages as the neat LC. Similarly, the disadvantage of large driving voltages can be avoided by curing under an applied voltage. The findings on our PSLCs without nanoparticles essentially confirm previous observations on the effect of curing conditions on the performance of PSLCs [32,33,34,35,36,37,38,39]. In addition, we succeeded in fabricating sufficiently small particles made of LiNbO_3_:Fe, which can be dispersed in the nematic liquid crystal E7 or in a polymer-stabilized liquid crystal based on E7. This marks an important progress. Under appropriate conditions, i.e., for polymerization in the isotropic phase under an applied DC voltage, the PSLC/NP composite shows decent driving voltages. Based on these promising findings, we expect, that ferroelectric nanoparticles with surface-grafted photo-initiators can in principle be poled with a sufficiently large DC field, covalently bound to a polymer network, and used to add polar properties to composites. An equivalent effect was observed previously in a polymer-stabilized antiferroelectric liquid crystal (PSAFLC) [31,32]. Curing under an applied DC field biased the electro-optic response of the PSAFLC, thereby enabling an optical storage effect or ferroelectric (as opposed to antiferroelectric) behavior of the PSAFLC [32]. Even more opportunities can be envisaged for LC composites that contain both nanoparticles and a polymer network. For example, poled PSLCs containing LiNbO_3_:Fe may facilitate optical addressing through the anomalous photovoltaic effect. Further studies, including the electro-optic response of PSLC/LC composites to DC voltages are required to explore such opportunities.

## Figures and Tables

**Table 1:** Composition of the samples studied. Components: E7, nematic LC mixture; LiNbO_3_:Fe, ferroelectric nanoparticles; EHA, 2-ethylhexylacrylat; RM 257, 1,4-Bis-[4-(3-acryloyloxypropyloxy) benzoyloxy]-2-methylbenzene; IRG 819, Bis(2,4,6-trimethylbenzoyl)-phenylphosphineoxide. All concentrations are given in percent by weight.

Composite	Concentration of the Components [% by Weight]				
	E7	LiNbO_3_:Fe	EHA	RM 257	IRG 819
Pure LC	100%	-	-	-	-
LC/NP dispersion	99%	1%	-	-	-
PSLC	89%	-	5%	5%	1%
PSLC/NP dispersion	88%	1%	5%	5%	1%

## Data Availability

All data that are relevant to this study are represented in this manuscript.
